# Energy-efficient waveform for electrical stimulation of the cochlear nerve

**DOI:** 10.1038/s41598-017-13671-y

**Published:** 2017-10-19

**Authors:** Marcus Yip, Peter Bowers, Victor Noel, Anantha Chandrakasan, Konstantina M. Stankovic

**Affiliations:** 1 Department of Electrical Engineering and Computer Science, Microsystems Technology Laboratories, Massachusetts Institute of Technology, 77 Massachusetts Avenue, Cambridge, MA 02139 USA; 2 Eaton-Peabody Laboratories, Department of Otolaryngology, 243 Charles Street, Massachusetts Eye and Ear, Boston, MA 02114 USA; 3 Program in Speech and Hearing Bioscience and Technology, Division of Medical Sciences, Harvard Medical School, 260 Longwood Avenue, Boston, MA 02115 USA; 4 Cochlear Implant Research Laboratory, 243 Charles Street, Massachusetts Eye and Ear, Boston, MA 02114 USA; 5 Department of Otolaryngology, Harvard Medical School, 25 Shattuck Street, Boston, MA 02115 USA

## Abstract

The cochlear implant (CI) is the most successful neural prosthesis, restoring the sensation of sound in people with severe-to-profound hearing loss by electrically stimulating the cochlear nerve. Existing CIs have an external, visible unit, and an internal, surgically-placed unit. There are significant challenges associated with the external unit, as it has limited utility and CI users often report a social stigma associated with prosthesis visibility. A fully-implantable CI (FICI) would address these issues. However, the volume constraint imposed on the FICI requires less power consumption compared to today’s CI. Because neural stimulation by CI electrodes accounts for up to 90% of power consumption, reduction in stimulation power will result directly in CI power savings. To determine an energy-efficient waveform for cochlear nerve stimulation, we used a genetic algorithm approach, incorporating a computational model of a single mammalian myelinated cochlear nerve fiber coupled to a stimulator-electrode-tissue interface. The algorithm’s prediction was tested *in vivo* in human CI subjects. We find that implementation of a non-rectangular biphasic neural stimulation waveform may result in up to 25% charge savings and energy savings within the comfortable range of hearing for CI users. The alternative waveform may enable future development of a FICI.

## Introduction

Sensorineural hearing loss (SNHL) is the most common sensory deficit and the most common congenital anomaly, affecting 360 million people worldwide^1^. The CI is a device that can restore hearing in people with severe-to-profound SNHL by electrically stimulating the cochlear nerve (CN), bypassing damaged or missing cells in the inner ear. The CI is the most widely-used neural prosthesis globally, with over 324,000 recipients as of December 2012^2^. The external unit includes a microphone, sound processor to digitize, analyze, and compress sound into coded signals, and transmitter to send data wirelessly to the internal unit via inductance. The implanted unit includes a receiver and stimulator seated in the skull, and an intracochlear electrode array. Electrical current stimulus is modulated by the received codes and delivered to the electrode array, triggering action potentials in the CN, which are interpreted by the brain as sound.

The external CI unit raises concerns with social stigma^3^, may lead to skin breakdown and pain at the site of transcutaneous coupling with the internal unit^4^, may be of limited use when playing sports, and cannot be worn when sleeping^5^, hence imposing a safety risk because of the user’s inability to hear warning alarms. Importantly, CIs can be permanently damaged by static electricity^5^. These limitations of the conventional CI motivate the development of an invisible, fully-implantable CI (FICI).

The limited energy storage capacity of a FICI requires low-power (<1 milliwatt total)^6,7^ sound processing and CN stimulation to enable operation from an implanted battery, wirelessly recharged only once daily. This low-power requirement is estimated assuming the FICI operates 12 hours/day, using a 5 g ultra-capacitor with an energy density of 5 watt-hr/kg, and that the power circuit is 50% efficient. To address these challenges, we tackle neural stimulation, which may account for up to 90% of the total system power budget. This high power consumption, relative to the power required for acoustic sensing, A/D conversion, and signal processing is due to the minimum amount of energy required to trigger an action potential in the AN, as well as power dissipated at the electrode-tissue interface.

The effectiveness of pseudo-monophasic waveforms has been studied^8–11^. However, this type of waveform is not used in practice, as a long discharge period is required before a subsequent excitatory pulse, preventing damage to neural tissue. In the case of biphasic waveform stimulation, the anodic phase quickly and accurately balances the charge. In some monophasic stimulation studies, growing exponentials have reduced threshold energy^8,9^. Others have concluded decaying exponentials can result in energy savings^10^. Wongsarnpigoon *et al*. examined square, growing-exponential, decaying-exponential, and rising-ramp waveforms, finding that no waveform was simultaneously charge-, energy-, and power-optimal^11^. The relative efficiency of these waveforms was further affected by the pulse width. One study, focusing on biphasic waveforms, suggests a truncated Gaussian cathodic phase followed by a rectangular anodic phase^12^, while another suggests both excitatory and recovery phases should be exponentially growing^13^. Both cathodic and anodic phases can elicit neural responses. In humans, it has been reported that the auditory system is more sensitive to anodic stimulation at suprathreshold levels^14,15^. Studies in other animals, such as guinea pigs, have reported that the cathodic phase is instead more efficient in eliciting a neural response^16^.

Triphasic stimulation is used in a limited number of current CIs, with the aim of diminishing effects of electrode interaction while maintaining charge balance, similarly to biphasic stimulation. Bahmer and Baumann^17^ studied the effect of triphasic and biphasic waveform stimulation of electrically-evoked compound action potentials (ECAP), considering the polarity of each. Their results show a symmetric triphasic waveform has the highest threshold and weakest ECAP response amplitude, while a biphasic (anodic phase first) waveform has the lowest threshold and strongest response amplitude. However, when considering power consumption of typical CI usage, threshold data do not provide a complete picture.

Our modeling *in silico* suggests an energy-efficient, biphasic-exponential waveform, having exponentially decaying cathodic and rectangular anodic phases, as an alternative to the traditional rectangular waveform. We have tested the charge and energy efficiency of a symmetric biphasic-exponential waveform *in vivo* by measuring loudness perception in humans chronically implanted with CIs.

## Results

### Energy-Optimal Waveform from a Genetic Algorithm

#### Genetic Algorithm Simulation Results for 25 μs, 50 μs, and 100 μs Phase Widths

**Figure 1:**
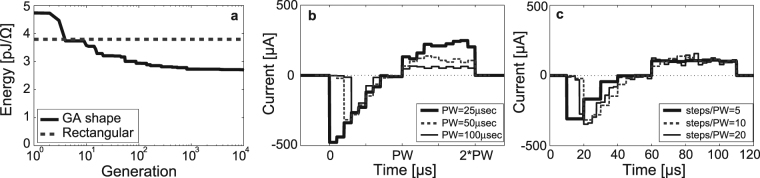
Genetic-algorithm generated waveforms. (**a**) Energy associated with GA-generated (solid line) waveform over 10^4^ generations compared to that of the rectangular waveform of same PW at action-potential generating threshold. (**b**) GA-generated waveforms after 10^4^ generations for PWs of 25 μs, 50 μs, and 100 μs (10 steps per phase), and (**c**) GA-generated waveforms after 10^4^ generations for (5, 10, and 20) steps per phase fixed at PW = 50 μs.

Genetic algorithm (GA) simulations were performed for pulses having 25 μs cathodic and 25 μs anodic phases (50 μs total pulse duration). After 10^4^ iterations, the GA waveform was approximately 28% more energy-efficient than the rectangular waveform, and it resembles an exponentially decaying cathodic phase, preceding a (roughly) rectangular anodic phase (Fig. 1a,b).

Phase widths (PW) used in today’s CIs may range from 10 μs to 200 μs^18,19^. The GA simulation was repeated for PW of 50 μs and 100 μs in order to investigate the impact of PW on optimal GA shape (Fig. 1b). The GA waveforms having PWs of 50 μs and 100 μs exhibit energy savings of 35% and 54%, respectively, compared to the corresponding rectangular waveform. For all three PWs, the resulting cathodic phase is a decaying waveform, with most of the charge delivery front-loaded in time and concentrated in a time window that lasts roughly 30 μs to 40 μs in duration. The 25 μs PW is non-zero for its entire duration, and as the PW increases, the charge spans only a portion of the PW. This suggests that there is a critical PW for neural stimulation and explains why there is a greater percent energy savings as the PW length increases. Finally, the shape of the anodic phase tends to a generally flat shape as the PW increases, as is the case for the 50 μs and 100 μs PW. The anodic phase is somewhat back-loaded for the 25 μs PW.

Assuming the shape of the cathodic phases are exponentially decaying, the estimated time constants are 9.0 μs, 6.9 μs, and 3.9 μs for the 25 μs, 50 μs, and 100 μs PW waveforms, respectively—time constants were determined from exponential equations fit to the data, using the built-in MATLAB function *cftools*: data points associated with times earlier than the peak absolute magnitude in the cathodic phase were not included in curve fitting.

Assuming the shape of the anodic phase to be rectangular in nature, its flatness Q can be described by the standard deviation divided by the mean value of the current across the anodic phase. Q of the anodic phase, as a comparison to the rectangular waveform, is quantified by the standard deviation of the data points divided by the mean value of the anodic phase current. This ratio is approximately 0.23, 0.19, and 0.12 for waveforms having PWs of 25 μs, 50 μs, and 100 μs, respectively.

#### Genetic Algorithm Simulation Results for Various Numbers of Genes

In the GA simulations so far discussed, the number of genes (time steps per phase) was held constant at 10. Simulations were performed for a PW of 50 μs, for (5, 10, and 20) steps/phase, over 10^4^ generations (Fig. 1c). In each case, the cathodic phase is decaying and front-loaded in nature, while the anodic phase is relatively flat. The energy savings are approximately 35% in each case when compared to the rectangular biphasic waveform. Properties of the CI stimulation hardware, such as bandwidth and settling time, limit the number of maximum steps, making 5 to 10 steps/phase (5 to 10 μs time-step) practical.

The estimated cathodic phase time constants are 11.9 μs, 13.9 μs, and 10.7 μs for waveforms having a steps/PW ratio equal to 5, 10, and 20, respectively, while the Q of the waveforms’ anodic phases are approximately 0.04, 0.23, and 0.21, respectively.

#### Comparison to Alternate Waveform Shapes

**Figure 2:**
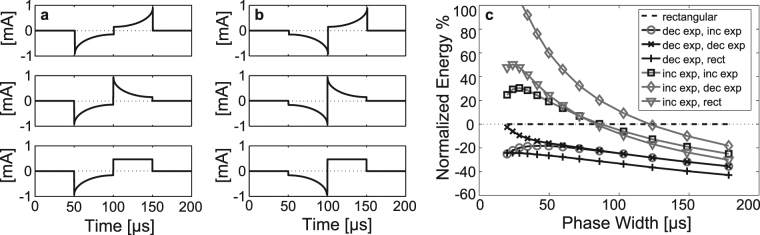
Representative biphasic waveforms with (**a**) decaying exponential cathodic phases, and (**b**) growing exponential cathodic phases. The anodic phases from top to bottom in each column are: exponential growing phase, exponential decaying phase, and rectangular phase. (**c**) Waveform energy, normalized to that of the standard biphasic rectangular waveform, is shown as a function of phase width for the waveforms in (**a**) and (**b**).

The exponential biphasic waveforms (exponential-decaying cathodic phase and rectangular anodic phase) were compared against common waveforms found in literature. The waveforms were either of decaying- or growing-exponential cathodic phase, with phases comprising 10 discrete time steps. In both cases, three possible anodic phases were examined: growing exponential, decaying exponential, and rectangular (Fig. 2a,b). The time constants of exponential phases were fixed to half of the PW. Percent energy expended compared to the rectangular biphasic waveform was measured as a function of PW from 20 μs to 180 μs (Fig. 2c) (PWs spaced logarithmically, with a total of 13 data points). All waveforms having a decaying-exponential cathodic (initial) phase are more energy-efficient than all of those having a growing-exponential cathodic phase across all PW (as well as compared to the rectangular biphasic waveform), and the most overall energy-efficient waveform has a rectangular anodic (secondary) phase, except at 20 μs. Above 50 μs, there was no difference in energy savings between the decaying-exponential cathodic phase waveforms having either a decaying- or increasing-exponential anodic phase. These results are in agreement with those of Jezernik and Morari^8^ showing that an exponential stimulation waveform—exponentially rising in their case—is increasingly energy-efficient as the pulse duration increases, when compared to a rectangular waveform.

The decaying-exponential cathodic, rectangular-anodic phase waveform has energy savings of 20–30% compared to the rectangular biphasic waveform below 50 μs, and energy savings of approximately 40% between 125 μs and 180 μs.

#### Exponential Time Constant of Cathodic Phase

**Figure 3:**
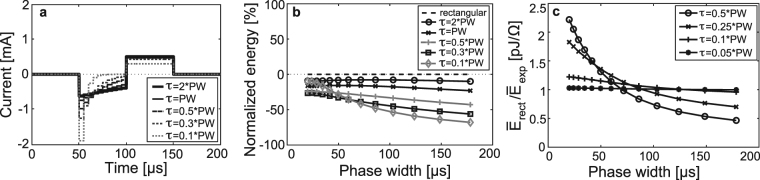
Comparison of pulse waveforms. (**a**) Biphasic CN stimulation waveforms having an exponentially decaying cathodic phase and a rectangular anodic phase equal in charge (to maintain charge balance). The time constant of the cathodic phase is varied. (**b**) The energy of the waveforms in (**a**) is compared to the energy of rectangular biphasic waveforms having the same PW and pulse duration. Energy is expressed as percent of the rectangular waveform energy, with negative percentages implying energy savings. (**c**) Monophasic exponentially decaying CN stimulation waveforms are compared to a rectangular monophasic pulse having a duration that is 20% of the PW. The ratio of energy of the rectangular pulse (${\mathop{E}\limits^{\bar{} }}_{{rect}}$) to the energy of the exponential waveform (${\mathop{E}\limits^{\bar{} }}_{{\exp }}$) is shown as a function of PW. Comparison is made for four exponential waveforms having unique time constants.

The effect of altering the time constant τ for the exponential-decaying cathodic, rectangular-anodic biphasic waveform was examined. Figure 3b shows the results of varying τ of the cathodic phase between τ = 0.1*PW and τ = 2*PW. The cathodic phase was discretized to 10 steps and the duty cycle was fixed at 50% (Fig. 3a). Shorter time constants result in a decrease in required energy for all examined phase widths, with the exception of the shortest time constant τ = 0.1*PW. In this case, below approximately PW = 60 μs its energy savings decrease significantly with decreasing PW, while above this PW it is the most energy-efficient waveform.

#### Comparison with Short Rectangular Pulses

The data from Fig. 3a suggest that as the exponential time constant decreases, the exponential waveform shape looks more like a short, rectangular pulse. It was therefore worthwhile to compare the decaying exponential waveforms to short rectangular pulses. For each cathodic PW examined, the phase was discretized to N number of steps, and the corresponding rectangular pulse to which each exponentially decaying waveform was compared had a width equal to the PW divided by N. Only monophasic stimulation was compared; the contribution of a secondary phase was not considered. A comparison of decaying exponential waveforms was made (Fig. 3c) (PWs spaced logarithmically, with a total of 13 data points). The waveforms had a phase width of PW and were discretized into 5 time steps, with rectangular pulses having a duration of PW/5. The PW ranged from 20 μs to 180 μs and the time constant was varied discretely from τ = 0.05*PW to τ = 0.5*PW. The ratio of energy of the short rectangle pulse to the energy of the exponential decaying waveform is close to 1 for the shortest time constant across all phase widths, as was expected. An increase in τ results in a ratio greater than one for short PWs and a ratio less than 1 for longer PWs. The time constant of τ = 0.5*PW is closest to that of the optimal GA waveform. In this case, the ratio of energy of the short rectangle pulse to the energy of the decaying exponential is much greater than 1 for PWs less than 70 μs. Energy efficiency is seen by the decaying exponential waveform for shorter PW, approximately less than 70 μs, when compared to a short rectangular pulse of duration PW/5. This may be explained by the strength-duration characteristic of electrically-evoked neural response that illustrates an inverse relationship between the stimulation duration and spike threshold. Therefore, with a very short duration of stimulation, the threshold for spike generation may increase to the point of negating any benefit from a short PW.

#### Neural Recruitment in the Genetic Algorithm

In order to investigate how neural recruitment might affect the shape of our energy-efficient waveform, 20 potentially-excitable neurons were included in the genetic algorithm and a stimulation waveform was generated. The nerve fibers were located at varying distances (randomized and evenly distributed) between 1 mm and 5 mm from the stimulating electrode and shared the same morphology. The waveform PW was 50 μs, having 10 steps per phase. The generated waveform is similar to that produced by the implementation of a single nerve fiber; the waveform has an exponentially-decaying cathodic phase (τ = 13.6 μs) and an anodic phase that is roughly flat (Q = 0.13).

### Loudness Tests in Human Subjects

The minimum amount of current required to elicit a percept was determined for each of 4 subjects; subject and subject testing characteristics are shown in Table 1. The threshold current corresponds to a loudness rating of zero. The threshold values are not the same for all subjects and the same loudness data points are not shared between every trial, so a direct comparison could not be made between the two waveform types at each loudness rating. Therefore, a linear mixed-effects regression model was developed to compare both injected charge and electrode energy (each calculated from current), as a function of loudness rating, between the exponential and rectangular waveforms.

**Table 1:** Summary of subject characteristics, stimulating electrode of cochlear electrode array, number of trials each subject performed for each waveform, and loudness scale chosen by subject.

Subject	Sex	Ear Tested	Electrode	Trials per Waveform	Loudness Scale*
A	Male	Right	7	3	0 to 50
B	Female	Right	7	4	0 to 10
C	Female	Left	7	4	0 to 25
D	Male	Right	9	2	0 to 25

*Loudness scale is the original scale chosen by the subject. All loudness scales were normalized to have a maximum of 25, with 8 to 22 being the range of interest (typical operation of device).

**Figure 4:**
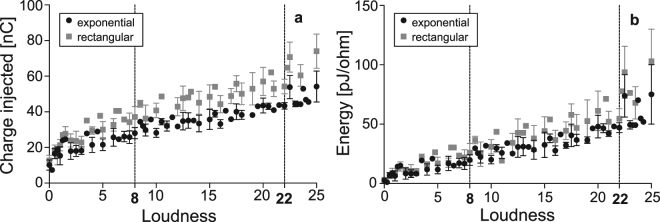
Results from loudness tests in 4 human subjects. Stimulation waveforms were a standard rectangular waveform and an energy-efficient waveform, having decaying exponential cathodic and growing exponential anodic phases. Each phase width was 54 μs, comprised of five time steps of equal duration. The time constant of each exponential phase was 25 μs. Stimulus was a pulse train of 1000 pulses/sec having a duration of 300 ms. (**a**) Charge injected [nC] and (**b**) electrode energy [pJ/ohm] of exponential waveform (decaying exponential cathodic phase, growing exponential anodic phase) and rectangular waveform per phase as a function of loudness; loudness range of 8 to 22 represents approximate range over which CI typically operates.

The dependent variable of the model was either charge or energy, while the independent variables were waveform type, loudness rating, and the waveform type/loudness rating interaction term. The models accounted for only data points having a loudness rating within the range of 8 to 22, which approximates the dynamic range of typical CI usage. The mean injected charge and the mean electrode energy, as a function of loudness rating, are shown in Fig. 4a and b, respectively. Mean values outside of the loudness rating range of interest are shown, but are not included in the models. Least square means (LSM) were calculated at a loudness rating of 15.38—the center of gravity (average) with respect to loudness of the data points within the loudness range of 8 to 22—to determine average charge and energy savings.

The use of the alternative exponential waveform results in a significant reduction in charge between loudness levels of 8 and 22 when compared to the standard biphasic rectangular waveform (p < 0.0001). The LSM charges of the exponential and rectangular waveforms are 36.3 ± 1.5 nC and 48.9 ± 1.5 nC, respectively, resulting in 25.8 ± 3.7% charge savings of the exponential waveform over the rectangular waveform. With respect to energy, the alternative waveform is significantly more efficient over the same loudness range when compared to the rectangular waveform (p < 0.0001). The LSM electrode energies of the exponential and rectangular waveforms are 33.8 ± 2.6 pJ/ohm and 45.40 ± 2.7 pJ/ohm, respectively, resulting in 25.5 ± 7.3% energy savings of the exponential waveform over the rectangular waveform.

There is a significant increase in charge efficiency of the alternative waveform, compared to the standard waveform, as loudness increases (p = 0.032); there is a reduction in charge of approximately 0.26 nC per unit loudness. With respect to energy, an increase in alternative waveform efficiency is seen as loudness increases when compared to the standard waveform, however this effect did not reach a statistical significance (p = 0.051); there is a reduction in charge of approximately 0.45 pJ/ohm per unit loudness.

## Discussion

The power budget of today’s cochlear implant is dominated by the stimulation power. A reduction in the energy required for stimulation of the CN fiber can translate into savings in the overall power savings of the device, lending toward the feasibility of a FICI. An alternative stimulation waveform, specifically biphasic and non-rectangular in nature, can provide a similar neural response and/or loudness perception that are produced by its rectangular counterpart, while requiring less charge and energy. An optimized biphasic waveform, generated by using a computational nerve-fiber model coupled to a GA is up to 28% more energy-efficient than the conventional rectangular waveform having a PW of 25 μs. For all PWs investigated, with no constraint on the anodic phase of the stimulation waveform, the cathodic phase of the energy-optimal GA waveform is decaying and front-loaded, while the anodic phase is typically flat. Furthermore, the effective portion of the cathodic phase of the energy-optimal waveform appears to have a duration of approximately 30 μs to 40 μs, regardless of the PW (assuming the PW is at least this long in duration).

The energy savings of the optimal GA waveforms were related to the savings associated with common waveforms found in literature, with respect to the standard biphasic rectangular waveform (Figs 2 and 3). In these comparisons, one manually-generated waveform shared the same general shape as that generated by the GA-generated waveform; the cathodic phase is exponentially decaying, while the anodic phase is perfectly rectangular. The energy savings of the GA-generated waveform (50 μs PW) and of the manually-generated waveform of similar shape are approximately 35% and 27%, respectively, which suggests that the unique shape of the GA waveform is not an artifact of the algorithm and is superior to that of the waveforms having perfectly exponential and/or rectangular phases.

Our data also show that decreasing the time constant of the exponential waveform, to a point, may increase energy-efficiency. However, at PWs below approximately 70 μs, decreasing the time constant can have a negative impact on efficiency. A short rectangular pulse, having a duration that is 20% that of the decaying exponential waveform PW, may be most energy-efficient at a PW greater than 70 μs. These findings suggest that a CI signal processor capable of switching between stimulation waveform shapes might provide energy savings.

The simplicity of a single nerve fiber in a homogenous and isotropic medium has several important limitations. First, it does not capture the complex anatomy and electrical properties of the cochlea as would otherwise be captured with a full 3-D patient-specific volume conduction model^20^, nor does it account for a neuron’s condition, and its distance and trajectory relative to the stimulating electrode. The effects of these factors on neural excitation have been studied in 3-D computational models^21–23^. Secondly, we use the model to predict fiber activation thresholds, while the psychophysical experiments concern loudness, and the connection between the two has not been investigated here. However, it was our intention to use the results from simulation using a single fiber model to predict a waveform worthy of psychophysical loudness testing in real patients.

To demonstrate its effectiveness, an alternative biphasic exponential waveform (decaying exponential cathodic, growing exponential anodic) was compared to a biphasic rectangular waveform *in vivo*. This alternative waveform was opted over the alternative waveform having an exponentially decaying cathodic phase followed by a rectangular anodic phase, as its phase symmetry ensures charge balancing. Subjective loudness experiments, using these two waveforms, were performed in humans. When the perceived loudness is within the comfortable range of hearing—being relevant because most of the power is dissipated within this region—charge savings of 25.81 ± 3.74% and energy savings of 25.46 ± 7.27% are seen with the implementation of the exponential waveform (calculated from LSM at a loudness rating of 15.38).

A GA-generated waveform (50 μs PW, 5 steps/phase) is 35% more energy-efficient compared to the rectangular biphasic waveform, approximately 10% greater efficiency than the waveform of the loudness experiments (54 μs PW, 5 steps/phase). This difference could be due to the nonuse of the rectangular anodic phase (due to charge balancing requirements), or might suggest that energy savings at loudness thresholds and at nerve-fiber action potential thresholds are dissimilar. Moreover, the GA algorithm may over-estimate the energy savings compared to real-world application. This 10% difference in savings can be seen in the comparison of waveforms in Fig. 2, where at 50 μs PW, the energy savings are approximately 9% higher for the exponential-rectangular waveform compared to the exponential-exponential. This suggests that the lower-than-expected energy savings in the loudness test can be attributed to the anodic phase compromise made to ensure charge balance.

It is worthwhile to note the perceptual observations made by the subjects during this experiment, although quality of the auditory sensation, other than loudness, was not the focus of the loudness test. Subjects B and C noted that the sound elicited by the rectangular waveform was lower in pitch than that elicited by the exponential waveform. Subject D noted that the exponential waveform sounded “shorter, more localized, focused”, while subject A noted the rectangular waveform was less “raspy”. It is important to take into account sound quality when considering a stimulation waveform, as the purpose of the device is to enable its user to distinguish sounds as effectively as possible. Allowing a subject to utilize an alternative waveform for an extended period could allow for determination of the effect of brain plasticity on the sound quality and speech recognition associated with an alternative stimulation strategy. For several months after CI implantation, reorganization of mechanisms within the brain allows users to achieve improved auditory perception^24^, a phenomenon that may also occur after the stimulation strategy of the device has been altered.

Our results offer a preliminary look into the reduction of CI power consumption by incorporating an alternative stimulation waveform. Further testing *in vivo* should be conducted to further support what has been shown here, including speech recognition tests to determine the functionality of such a waveform in comparison to the biphasic rectangular waveforms currently employed in today’s CI.

## Methods

### Simulation of nerve fibers and stimulator-electrode-tissue interface

We focus on minimizing the waveform energy, which is the energy delivered to the electrode, ignoring any wasted overhead power in the stimulation circuitry. As the efficiency of delivering power to the electrode approaches 100% with advancements in technology, the electrode energy becomes the theoretical minimum amount of delivered energy required of the stimulator. This energy is defined as the energy per unit resistance in the electrode over the phase width, as shown in equation 1, where *E*_*elec*_ is the total waveform energy, *R*_*elec*_ is the electrode resistance, and *I(t)* is the current of the stimulation waveform.

1 $${\bar{E}}_{elec}=\frac{{E}_{elec}}{{R}_{elec}}={\int }_{\langle PW\rangle }{I}^{2}(t)dt$$

In the case of current source-based stimulators, the total charge delivered *Q*_*tot*_ is the more relevant quantity, and is calculated as the current integrated over the PW; for current source based stimulators, it can be assumed that the supply voltage is fixed, therefore the total energy required for stimulation scales with total charge. This relationship is shown in equation 2. If the excitatory phase of the stimulation is of the same shape as the recovery phase, the total charge over the entire pulse is simply twice the charge calculated for the individual phases. If the shapes are different, and not simply mirrored, the charge for each phase must be calculated separately and added. This applies for energy calculations as well.

2 $${Q}_{tot}={\int }_{\langle PW\rangle }I(t)dt$$

**Figure 5:**
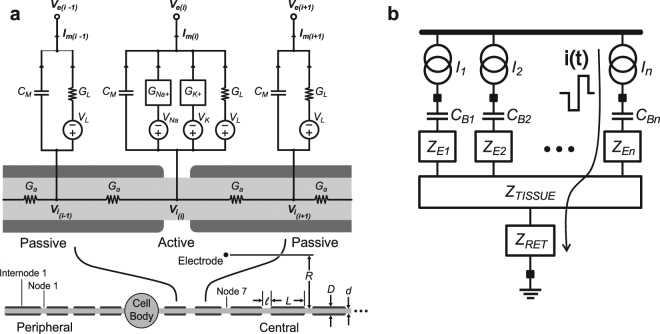
Nerve fiber and electrode portions of circuit model for generating action potentials using energy-efficient waveform for CN stimulation. (**a**) Circuit model of a single mammalian myelinated nerve fiber^20^. *I*_*m*_ is the membrane current at the node of Ranvier (modeled actively by the non-linear conductances describing the kinetics of the sodium and potassium ion channels, *G*_*Na+*_ and *G*_*K+*_, respectively)^25,26^. *V*_*i*_ and *V*_*e*_ are the intracellular and extracellular potentials, respectively. *V*_*NA*_ and *V*_*K*_ are sodium and potassium resting potentials, respectively. *V*_*L*_ is the leak reversal potential. The passive, leaky insulator *G*_*L*_ accounts for the internode membrane current, and *G*_*a*_ is the axial conductance. The membrane capacitance is denoted *C*_*M*_. The point-source electrode is located at the 7^th^ node central to the peripheral end of the fiber. The nerve fiber comprises 21 nodes and 22 internodal segments; both the peripheral process and the central axon are terminated by myelinated internodal segments. Fiber dimensions are the distance from the electrode to fiber tissue *R*, nodal and internodal lengths *l* and *L*, respectively, and nodal and internodal diameters *d* and *D*, respectively. (**b**) Electrode portion of model illustrates the stimulation current *i(t)* path, originating from the n^th^ current source *I*_*n*_, continuing through an optional DC blocking capacitor *C*_*Bn*_ (usually 100’s of nF) to the intracochlear electrode. *Z*_*E*_, *Z*_*TISSUE*_, and *Z*_*RET*_ are electrode, tissue, and return impedances, respectively.

A computational model of a mammalian myelinated CN fiber was utilized in order to find energy-optimal waveforms effective in generating action potentials within the CN; for current source based stimulators, charge efficiency and energy efficiency are equivalent, assuming the same stimulator supplies the voltage. The single-fiber model is a Hodgkin-Huxley type discrete cable model (Fig. 5a). It is assumed that fiber trajectory is in the radial direction from the point of innervation along the organ of Corti to the perikaryon in Rosenthal’s canal. The nodal characteristics are those of Frijns^25^, which were adapted from Schwarz and Eikhof^26^. The model, including cellular morphology, is described in detail by Whiten^20^ (sections 2.4.2 and 2.4.3). These nerve-fiber simulations are useful for determining the energy efficiency and effectiveness of various waveforms on evoking an action potential in a single nerve fiber, and do not reveal the influence of the waveform shape on auditory perception, which can be addressed through *in vivo* tests, such as the human psychophysical loudness tests included in this study.

The CN fiber model was coupled to a stimulator-electrode-tissue interface model, including the stimulator’s *n* current sources (Fig. 5b), electrodes, and target neural tissue. The electrode-to-nerve distance (*r* of Equation 3) was set at 1 mm. A high-frequency electrode model accounts for the power dissipated at the electrode-solution interface. It is comprised of a double-layer capacitance (5 nF–50 nF) at the interface and an electrode contact resistance of 2 ﻿kΩ–10 kΩ.

The spread of the current through the tissue can be simplified by the assumption that the cochlear tissue is homogeneous and isotropic^27^, having a resistivity of *ρ*_*e*_ = 300 Ω·cm. The stimulation current *i(t)* generates a voltage in the neural tissue at a distance *r* from the point-source electrode, which can be calculated as in equation 3^28^.

3 $${V}_{e}(t)=\frac{{\rho }_{e}i(t)}{4\pi r}$$

The voltage *V*_*e*_ *(t)* is the input (extracellular potential) applied to the nerve fiber model, taken from the potential field estimated along a fiber track at node or internode *i*, for a given stimulus current applied to the most peripheral internode (Fig. 5a). The voltage may trigger an action potential, depending upon the shape and the strength of the stimulus. Membrane capacitance *G*_*m*_ and leaky membrane conductance *G*_*L*_ are functions of either the nodal (*d*, *l*) or internodal dimensions of the fiber (*D*, *L*) as described by Whiten (section 2.7.2). The number of nodes and internodes are 21 and 22, respectively. The current is collected from the tissue via a common extracochlear return electrode. The impedance of the return electrode *Z*_*RET*_ is much smaller than *Z*_*Ei*_ electrode impedance and is therefore neglected.

The stimulating electrode is positioned at the 7^th^ node central to the peripheral end of the fiber, and two nodes from the cell body in the same direction (Fig. 5a). The peripheral process and central axon are terminated by an internodal segment to avoid an inappropriately high conductivity that would otherwise exist at an unmyelinated termination.

### Energy-Optimal Waveform from a Genetic Algorithm

The complexity and non-linearity of the CN fiber computational model prevents determination of an analytical solution for the energy-optimal stimulus waveform. We therefore used a genetic algorithm (GA) to heuristically determine an optimal waveform shape^12^. A biphasic stimulation waveform was optimized using such a method, incorporating stimulation parameters targeted for CI using the single-fiber model described above.

Genetic algorithms attempt to find solutions to complex optimization problems in a way that is similar to the process of natural evolution. The genetic algorithm begins with an initial population of 50 waveforms, each discretized to 20 time steps (i.e., 20 genes). The cathodic and anodic phases of the waveform each contain 10 time steps, providing enough degrees of freedom to approximate any arbitrary waveform shape, while being a reasonable level of discretization for practical simulation circuits limited by bandwidth and settling. No constraint was placed on the shape of either the cathodic or anodic phase, so that they may be co-optimized. The magnitude of each gene of the initial population solutions was randomly selected. The waveforms of each generation were applied to the nerve fiber model and the fitness quotient (e.g., energy of the waveform) was calculated. A severe energy penalty was applied if no action potential was initiated. If a waveform is rejected for not initiating an action potential, a new waveform of the same shape and different amplitude may still be generated. The fittest waveform of each generation was recorded and the 10 fittest parent waveforms generated offspring for the subsequent generation. To increase the probability of escaping any local minima, we included random gene mutation (i.e. scaling) in each iteration, with a variance of 0.025 and mean of 1 from one cycle to the next. After many iterations (e.g., 10^4^), the output is the stimulation waveform having the lowest energy that is able to initiate an action potential. These simulations were performed for phase widths of (25, 50, and 100) μs, with the duty cycle fixed at 50%. The number of time steps was also considered; GA simulations were performed at (5, 10, and 20) steps/phase.

The optimal GA waveforms were compared against common waveforms found in literature. One set of waveforms was of exponentially decaying cathodic phase and a second set was of waveforms having growing exponential cathodic phases. Each set had the following possible anodic phases: growing exponential, decaying exponential, or rectangular. Finally, GA simulations were generated while the time constant of the decaying exponential cathodic phase was fixed and the overall phase width was varied. The time constant for the unique simulations ranged from τ = 0.1*PW to τ = 2*PW.

### Loudness Test in Human Subjects

Four adult humans subjects, who have been implanted with a HiRes90 k device from Advanced Bionics (AB) for at least 18 months, were tested (CI performance tends to plateau in postlingually deafened adults by 6 months^29^). The testing was approved by the Massachussets Eye and Ear (MEE) Human Studies Committee (IRB protocol #94-01-003) and the MIT Committee On the Use of Humans as Experimental Subjects (COUHES), and performed at the Cochlear Implant Research Laboratory at MEE. All experiments were performed in accordance with relevant guidelines and regulations. Informed consent was obtained from all subjects. Custom interface hardware for the AB system, which bypasses the wearable speech processors and directly controls the implanted current stimulators, was used.

A psychophysical subjective loudness perception test was performed using two biphasic waveforms: a standard rectangular waveform and an energy-efficient waveform, having decaying exponential cathodic and growing exponential anodic phases. The motivation for this test, as opposed to simply determining the charge and energy required for auditory threshold, was that the stimulus levels provided by the CI during regular usage are well above the threshold of hearing. Moreover, savings at high power levels are more beneficial than savings at the threshold of hearing.

The choice of non-rectangular waveform was based on results of the genetic algorithm used to determine an energy-optimal waveform. Although the results suggest that a waveform having a decaying exponential cathodic phase followed by a rectangular anodic phase is most energy-efficient, the phase symmetry of the tested alternative waveform ensured charge balance; the anodic phase shape was that of a growing exponential. Each phase width was 54 μs, comprised of five time steps of equal duration. The time constant of each exponential phase was 25 μs. The stimulus was a pulse train of 1000 pulses/sec having a duration of 300 ms.

Threshold current was first determined by performing a three-alternative forced choice test (2 down, 1 up). The stimulation amplitude was increased from threshold to just beyond the subject’s maximum comfort level in 50 μA increments. The subjects were asked to rate the loudness at each stepped current level. Each subject chose a loudness scale with which he or she could judge comfortably; absolute scaling of loudness is a widely used method because subjects rate based on a single, internal, presumably consistent scale that is closely aligned with loudness, as opposed to other sound qualities that may accompany loudness^30,31^. Test specifics for each individual, including chosen loudness scale, are shown in Table 1. The injected current as a function of rating was rescaled for subjects C and D to correspond to a 0 to 25 loudness rating scale. Within this normalized scale, a loudness of 8 is representative of the minimum level the subject could judge comfortably, and 22 represents the loudest sound not causing discomfort. Each subject performed a practice trial for both the threshold and loudness test. The number of non-practice trials performed for each waveform and both test types are listed in Table 1. Non-practice trials were performed in a pseudo-random order.

The primary and secondary outcome measures were the total energy and total charge, respectively, per cathodic phase required to elicit certain loudness. Due to the nature of these experiments ([a] subjects have different threshold currents, and [b] some loudness data points are not shared between all subjects for all trials), a direct comparison between waveforms could not be performed at each loudness rating.
